# Multidisciplinary Effort to Drive Precision-Medicine for the Future

**DOI:** 10.3389/fdgth.2022.845405

**Published:** 2022-05-02

**Authors:** Dewei Kong, Haojie Yu, Xueling Sim, Kevin White, E. Shyong Tai, Markus Wenk, Adrian Kee Keong Teo

**Affiliations:** ^1^Stem Cells and Diabetes Laboratory, Institute of Molecular and Cell Biology, Agency for Science, Technology and Research (A*STAR), Singapore, Singapore; ^2^Dean's Office, Yong Loo Lin School of Medicine, National University of Singapore, Singapore, Singapore; ^3^Department of Obstetrics and Gynaecology, Yong Loo Lin School of Medicine, National University of Singapore, Singapore, Singapore; ^4^Department of Biochemistry, Yong Loo Lin School of Medicine, National University of Singapore, Singapore, Singapore; ^5^Precision Medicine Translational Research Programme (TRP), Yong Loo Lin School of Medicine, National University of Singapore, Singapore, Singapore; ^6^Saw Swee Hock School of Public Health, National University of Singapore and National University Health System, Singapore, Singapore; ^7^Genome Institute of Singapore, A*STAR, Singapore, Singapore; ^8^Department of Medicine, Yong Loo Lin School of Medicine, National University of Singapore, Singapore, Singapore

**Keywords:** precision medicine, big data, omics technologies, data collection, data processing, results interpretation, metabolic disease, healthcare

## Abstract

In the past one or two decades, countries across the world have successively implemented different precision medicine (PM) programs, and also cooperated to implement international PM programs. We are now in the era of PM. Singapore's National Precision Medicine (NPM) program, initiated in 2017, is now entering its second phase to generate a large genomic database for Asians. The National University of Singapore (NUS) also launched its own PM translational research program (TRP) in 2021, aimed at consolidating multidisciplinary expertise within the Yong Loo Lin School of Medicine to develop collaborative projects that can help to identify and validate novel therapeutic targets for the realization of PM. To achieve this, appropriate data collection, data processing, and results interpretation must be taken into consideration. There may be some difficulties during these processes, but with the improvement of relevant rules and the continuous development of omics-based technologies, we will be able to solve these problems, eventually achieving precise prediction, diagnosis, treatment, or even prevention of diseases.

## Introduction

Precision medicine (PM) refers to the medical model that considers individual gene variability, environment, and lifestyle to achieve targeted treatment for specific groups of patients and diseases (1). It is not a new term and concepts similar to PM have surfaced since ancient times (2). As early as the last century, the idea of personalized medicine, a concept similar to PM but more focused on the individual, has come to attention (3). In the 1960s and 1970s, people have already realized the importance of providing the right drug for the right patient (4). The introduction of drug metabolizing enzyme cytochrome P450 (CYP450) and the discovery of human epidermal growth factor receptor 2 (*HER2*) gene were considered two milestones of PM (5). Later on, the completion of the Human Genome Project (HGP) in 2003 then opened the door to modern PM (6). Nowadays, various omics data, including genomics, epigenomics, transcriptomics, proteomics, metabolomics, and microbiomics, together with data beyond omics, including social, behavior, and environmental factors have been taken into consideration for the field of PM (7). Further details on PM can be found in the book “Toward Precision Medicine: Building a Knowledge Network for Biomedical Research and a New Taxonomy of Disease” written by the National Research Council of the National Academics in 2011 (8).

In the past one or two decades, countries across the world have successively implemented PM programs (Table 1). Many PM programs are now aimed at generating a large database that contains comprehensive health information of the participants. With the rapid development of big data and artificial intelligence (AI), researchers can then analyze these data to generate biomarkers for predicting, diagnosing, and even possibly treating specific disease subtypes. Therefore, the collection, processing, and interpretation of these data are becoming more and more important. In this article, we summarize current PM programs in different countries as well as some international programs, with a focus on the PM program at the National University of Singapore (NUS) in Singapore. We discuss the multidisciplinary expertise required, and how our collective efforts can come together to facilitate the vision of realizing PM at NUS. Last but not least, we discuss some of the existing challenges facing PM programs and possible ways of addressing them so as to realize their potential for the future of PM.

**Table 1:** A non-exhaustive list of PM programs in different countries and regional or international PM programs.

**Country**	**Name of the program**	**Type of data collected**	**Number of participants**	**Year**	**References**
Australia	Australian Genomics	Genomic and clinical data	5,000 (recruited)	2016	(9)
Brazil	The Brazilian Initiative on Precision Medicine (BIPMed)	Genomic data	NA	2015	(10, 11)
Canada	Genome Canada	Genomic and proteomic data	NA	2000	(12)
China	China's precision medicine initiative	NA	NA	2015	(13)
France	Genomic Medicine France 2025	Genomic data	10,000 (planned)	2015	(14)
Germany	The Centers for Personalized Medicine	NA	NA	2019	(15)
Japan	Cancer Genome Screening Project for Individualized Medicine in Japan	Genomic, transcriptomic, and proteomic data	20,000 (recruited)	2015	(16)
Singapore	Singapore's national precision medicine (NPM) program	Genomic, clinical, and lifestyle data	Around 1,000,000 (planned)	2017	(17)
U.K.	100,000 Genomes Project	Genomic data	85,000 (recruited)	2013	(18)
U.S.	Precision Medicine Initiative (All of Us)	Participant-provided information, Electronic Health Records, physical measurements, and biospecimens	1,000,000 or more (planned)	2015	(1, 19)
Denmark, Iceland, Estonia, Norway, Finland, Sweden	Nordic Society of Human Genetics and Precision Medicine	NA	NA	2018	(20)
International	International HundredK+ Cohorts Consortium (IHCC)	NA	100,000 or more (planned)	2018	(21)
International (mainly European countries)	The International Consortium for Personalized Medicine (ICPerMed)	Molecular profiling, medical imaging, and lifestyle data	NA	2016	(22, 23)
European Union countries, U.K., Norway	1+ Million Genomes Initiative (1+MG)	Genomic data	1,000,000 genomes (planned)	2018	(24)
International	The Global Alliance for Genomics and Health (GA4GH)	Genomic and phenotypic data	NA	2013	(25)

*NA indicates that the data could not be found*.

## Overview of Global PM Programs

PM has developed rapidly in the last one or two decades. According to Precedence Research, the global PM market is worth over USD $59.5 billion in 2020 and is expected to reach USD $141.33 billion by 2027 (26). Launched in 2015, the Precision Medicine Initiative became the largest longitudinal study in the history of the United States (U.S.), aiming to study diseases by taking the variability in gene, environment, and lifestyle into consideration (1). As the world's largest developing country, China implemented its PM program named the China's Precision Medicine Initiative in 2015 (13). European countries have also made a lot of efforts for PM, implementing a series of programs. France implemented the Genomic Medicine France 2025 in 2015, which aims to integrate genomics into the health care system and establish a genomic medicine industry (14). Germany established the Centers for Personalized Medicine in 2019, with the first focus on precision oncology (15). The United Kingdom (U.K.) has also launched the 100,000 Genomes Project in 2013, aiming to perform genome sequencing on people with rare diseases and cancer (18). In Singapore, the National Precision Medicine (NPM) program was launched in 2017, with a 10-year plan aimed at accelerating biomedical research, improving health outcomes, and enhancing opportunities for economic value across sectors (17). With Singapore's diverse demographics, the NPM program will likely fill in the gaps of inadequate PM data in Asia. We have now summarized a list of national and international PM programs in Table 1.

Besides PM programs initiated by individual countries, some international PM programs have also been established, such as the International HundredK+ Cohorts Consortium (IHCC) (21), the International Consortium for Personalized Medicine (ICPerMed) (22, 23), 1+ Million Genomes Initiative (1+MG) (24), and the Global Alliance for Genomics and Health (GA4GH) (25). These international programs are designed to make up for possible shortcomings of individual programs, bringing together the strengths of different individuals to work together for a common goal.

## PM in NUS, Singapore

**Figure 1:**
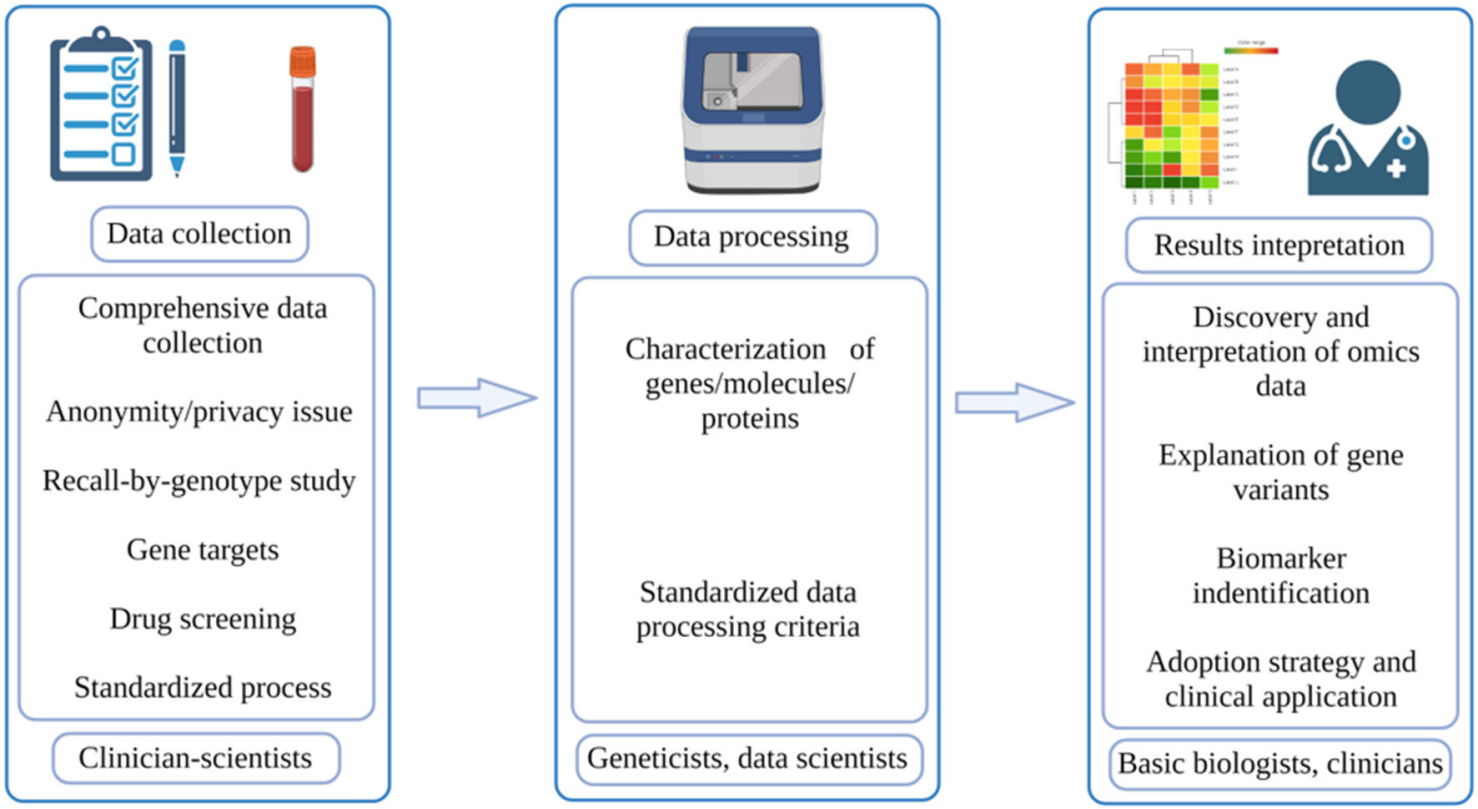
Workflow of PM. The typical workflow of PM involves three parts: data collection, which is done by clinician-scientists; data processing, which is done by geneticists and data scientists; results interpretation, which is done by basic biologists and doctors (created with BioRender.com).

As the oldest and most established university in Singapore, the NUS has also launched its own PM program. Led by Profs. Markus Wenk and E Shyong Tai, our PM translational research program (TRP) is comprised of clinician-scientists, population geneticists, genomics and proteomics scientists, bioinformaticians, and cell and molecular biologists coming together to facilitate the necessary pipeline for PM (Figure 1). With this multidisciplinary team, our PM TRP in NUS aims to leverage upon our access to a large and ethnically-diverse population, and use -omics datasets combined with deep mechanistic capabilities to develop targeted therapeutic outcomes for our patients. We want to help stratify populations based on disease mechanisms, identify and validate new therapeutic targets, and then translate these findings to the clinic.

We envision a close collaboration between clinician-scientists, geneticists, data scientists, and basic biologists who can facilitate the passage of clinical insights to disease stratification based on mechanisms and finally to PM right at the bedside (Figure 1). The unique demographic structure of Singapore certainly makes this program even more meaningful as our findings can potentially be applied across Asia, benefitting the Chinese, Malay and Indian ethnicity in our Asia-Pacific region. With the establishment of the National Electronic Health Record (NEHR) at the Singapore national level, we look to tap into the wealth of Asian genetic information, accumulate population-wide omics datasets and perform Asian-specific disease mechanistic studies. Together, our multidisciplinary team within the NUS PM TRP will tackle PM in metabolic disease(s) in the first instance.

## Different Phases of PM and Potential Challenges

According to McCarthy and Birney, to truly achieve PM, researchers need to collect data from a wide range of populations, take into account both genetic and non-genetic factors, and stratify/quantify risk levels rather than simply classifying them as low risk or high risk (27). As for its expected results, Prosperi et al. viewed that PM can achieve three goals in a continuous manner: disease diagnosis, disease prediction and prevention, and disease treatment (7). In the actual execution process, PM can be divided into three phases: data collection, data processing, and results interpretation (Figure 1). The quality of these processes will directly determine the final outcomes of PM. In the paragraphs below, some potential challenges regarding these phases will be discussed.

### Data Collection

#### Comprehensive Data Collection

Comprehensive data collection of a targeted population is the first problem all PM programs have to solve. Many current PM programs aim to recruit large numbers of targeted populations to build a comprehensive database. For example, Singapore's NPM program recruited 10,000 healthy people in its first phase and plans to recruit 100,000 healthy individuals and 50,000 patients with specific diseases in its second phase. This program will collect genomic data and other types of health data that can synergistically provide a comprehensive picture of a person's health status. Many other PM programs also collect a lot of data about a person's health. Therefore, big data will certainly form the core of PM. The creation of biological databases and biobanks that provide health information can undoubtedly help this process. With large, comprehensive databases, researchers can share the data with each other and obtain the information that they need more readily.

#### Problems During Data Collection and Possible Solutions

While the establishment of these databases will undoubtedly promote the development of PM, the data collection process inevitably raises some concerns. One of the major concerns is the anonymity or privacy of the participants. Participants or patients with a specific disease voluntarily provide data but the data must be kept anonymous during the study. De-identification is the process that removes the information that is directly or indirectly related to the participant (28). This process is essential to protect the privacy of the participants. However, achieving complete data anonymity is technically difficult, and is not desirable for some PM programs that involve clinical care (28). Therefore, it is understandable that society will have concerns and distrust over medical data security and how it can be properly guarded. This will inevitably retard the data collection process, and thus impede the development of PM to some extent. The continued development of AI technologies may eventually provide a solution to the security and privacy of the data. Briguglio et al. described a machine learning (ML) method to ensure patient privacy via encryption (29). Besides, Warnat-Herresthal et al. developed swarm learning method that helps data integration on the premise of confidentiality (30). By reducing direct human involvement, AI may provide some level of protection on data security and privacy.

In a type of targeted clinical study named recall-by-genotype (RbG) study, participants with defined genetic variations are specifically recruited (31). While participant anonymity and privacy become harder to protect due to the targeted recruitment of a small number of individuals, this study design is more cost-effective than other approaches as the research can be conducted in-depth on a small number of participants (31). Importantly, the RbG study can facilitate the elucidation of causal relationship between genetic variation and human physiology. Further biochemical and human cellular studies performed on these participants can then provide more definitive data that can eventually aid in the decision-making matrix for the implementation of PM.

#### Appropriate Choice and Interpretation of the Model System

With the availability of sequencing-based technologies and -omics methods, data collection, processing, and interpretation for genomics, epigenomics, transcriptomics, proteomics, metabolomics, and microbiomics datasets are seemingly straightforward. However, the major difficulty is likely to be the choice and availability of the correct human cell type at the correct developmental state for -omics analyses. For any given gene variant of interest, the cell type in which it is studied will result in a different interpretation as the transcriptional network and/or signaling pathways affected can be cell type- and cell state-specific. Gene targets may differ in different cellular contexts. The use of readily available human primary cells such as blood, skin, fat, or muscle cells vs. human induced pluripotent stem cell (hiPSC)-derived cell types such as brain, liver, or pancreas cells will also need to be determined carefully. Introduction of gene correction of variants in these human cell types for -omics data collection is also a current possibility. Last but not least, data collection from properly-determined cellular functional assays will also be critical for ascertaining genotype-phenotype causality.

In addition to finding population-specific targets using various omics technologies, another important aspect of PM is to perform population/genotype-based drug screening. Animal models and immortalized cell lines are two commonly used methods for drug screening (32). However, animal models often have interspecies differences (33), and many successful experiments in animal models do not translate well into clinical trials (32). Immortalized cell lines may also exhibit some differences compared to primary human cells (32). In recent years, the hiPSC-derived organoid model has become a popular model for studying the effects of human gene variants on cellular development and function (34–36). These cells can be used for population/genotype-based drug screening.

Besides the type of human cell and their cell state, the scale at which data needs to be collected for PM efforts will also need to be immense. Following which, there is also a need for a standardized process in which data is collected so that they can be processed in a similar manner for consistent and reproducible interpretation. In this regard, inter-laboratory comparison trials (or ring trials) will also be required to demonstrate the robustness of data collection processes that will ultimately lead to consistent and reproducible data. The use of pre-determined targeted gene/molecular panels instead of unrestricted-omics data collection may also be easier to synchronize data processing efforts.

### Data Processing

Data that has been collected will need to be processed and analyzed in detail by researchers to stratify populations into subgroups with different risk levels, so that novel biomarkers can be discovered and used in disease prediction, diagnosis, and treatment. Currently, there is still a need for detailed characterization of the roles of many human genes/molecules/proteins, development of a comprehensive human gene regulatory map, and catalog of cellular programs. This will be the basis in which the effects of gene variants can be compared against. Functional assays to determine the roles of many human genes in various cell types remain to be established. Therefore, many discoveries are still not well-translated into clinical strategies. Besides, the current complex biological systems without sufficient mechanistic understanding make it difficult to predict individual disease conditions and outcomes properly (37).

Although the development of AI and novel algorithms has greatly helped in data processing, insufficiently standardized data processing criteria may still render results to be irreproducible or unreliable. This is particularly evident in some omics datasets. On one hand, researchers would want to include more variables to improve the accuracy of their prediction (37). But on the other hand, the large number of variables itself greatly increases the difficulty of prediction (37). Striking a fine balance between these two parameters will be crucial in managing successful PM outcomes.

### Results Interpretation

Following data collection and processing, correct and appropriate data interpretation is likely the hardest and the most important part in order to realize its clinical value for PM. Omics data is understandably an important part of PM and researchers will typically focus on capturing invariant genomics data at the population level before expanding the scope to other dynamic omics datasets such as proteomics and metabolomics.

The discovery and interpretation of omics data currently present a major challenge for PM. Firstly, there is a large number of unknown regions in the human genome that impedes the decoding of the genome, thus hindering the development of PM. Whole genome sequencing (WGS) will readily facilitate deciphering of the coding region of the human genome but it only accounts for about 2% of the whole genome (38). The remaining 98% of the genome is thought to be associated with non-coding RNAs, regulatory sequences, and other sequences that might play a role and contribute to human disease. However, the function of most of these sequences and genomic interactions remain unclear to date. This is indeed a huge challenge for the realization of the utility of PM. Therefore, there is a need for more efforts focused on deep mechanistic studies at the cell and molecular level, to complement all the high throughput data collection. One current problem is the existence of variants of uncertain significance (VUS). VUS refers to the genetic variation that has been identified but without a clear function. It is currently almost impossible to track and interpret all the VUSs in patients (38). Hence, the presence of VUSs makes it even harder to accurately analyze the detailed mechanisms of some human diseases. For example, breast cancer 1 (*BRCA1*) and *BRAC2* are two widely studied genes. While lots of studies have been done on them, they still produce VUSs regularly (39). Much more human biology remains to be researched upon. In recent years, the growing interest in RNA biology and the use of bulk and single cell transcriptomics analyses may partly help to solve this problem.

The next concern facing PM is the appropriate explanation of these gene variants. With the rapid development of omics technologies, we can get the correct and precise localization of these gene variants. However, in many instances, their meaning in a particular population or group of individuals remain totally unclear. Genes are related and integrated functionally. Therefore, in different biological contexts, the effect of the same gene mutation might be different (40). In the same vein, the meaning of proteomic or metabolomic signatures in a stratified population will also need to be understood. These uncertainties increase the difficulties of interpreting the effects of gene variants. In order to do so, these gene variants and molecular differences will need to be studied in relevant animal/cell models in detail so as to interpret the meaning and apply the findings appropriately for stratified medicine. Thus far, some omics data have been successfully applied in PM. For instance, breast cancer is a highly heterogeneous disease where different disease subtypes have different pathogenesis and will need different treatment methods (41). Currently, breast cancer has been successfully divided into different subtypes, and its molecular mechanism(s) have been studied to a certain extent (41). This will help the targeted treatment for different patients. Familial hypercholesterolemia (FH) is another successful example. FH is a genetic cardiovascular disease that can increase the risk of having premature atherosclerotic cardiovascular disease if not treated properly (42). To address FH, Identification Methods, Patient Activation, and Cascade Testing for FH (IMPACT-FH) Study, a multi-disciplinary study, was established to help to identify individuals with high risk of having FH (43). In addition, the use of omics data to identify biomarkers to accurately predict disease occurrence and progression is also an important aspect of PM. Li et al. successfully identified oligoadenylate synthetases-like as a gene biomarker in differentiating non-influenza- and influenza-based acute respiratory infection using bioinformatics tools (44). Therefore, biomarkers identified by omics technologies might provide a good way to predict diseases at the population level. With the continuous development of various technologies, strategies, and in-depth mechanistic studies into unique gene variants and/or molecular differences between population groups, we can expect more successful applications for PM in the near future.

Upon successfully interpreting the meaning of gene variants and molecules linked to a particular disease, the adoption strategy is also critical for PM. First, the data collected, processed (e.g., data transformation), and interpreted needs to be packaged (e.g., presented with appropriate visualization) in a meaningful way that clinicians can understand. These test results must remain compliant with a myriad of regulatory constraints, integrating across data, lab, product, and business practice. Thereafter, clinicians need to be able to make the test accessible to patients and interpret the test for them. The test results need to be customizable and adapted to the needs of patients as patients may have difficulties understanding basic medical information and may not be ready to receive unexpected results (45). Appropriate decision support also needs to be in place during this stakeholder engagement. Clinically, Qian et al. developed a multidisciplinary therapy strategy of precision medicine (MDTS-PM) to apply PM, covering the whole range of services from diagnosis to treatment (42). In the field of tumor biology, Qian et al. opined that patients who have reliable gene test results, complex refractory tumors and drug resistance, are suitable for PM, and appropriate multidisciplinary therapy (MDT) strategy can have maximum benefits to those patients and clinicians (46).

## Discussion

The implementation of PM programs has indeed attracted extensive attention since its conception. In general, PM can be regarded as the use of technical means to analyze big data of healthy populations and patients with specific disease(s) to achieve precise prediction, diagnosis, and treatment of specific subgroups of patients. Unlike personalized medicine, which pays more attention to individuals, PM mainly focuses on the stratification of patients with seemingly similar disease, e.g., diabetes, into subpopulations of patients based on disease mechanism to develop targeted therapeutic strategies (3). In recent years, with the development of big data and AI, the establishment of PM programs in various countries has also developed rapidly. This article has summarized the PM programs in different countries, with a focus on the PM TRP of NUS. Drawing on the strengths of clinicians, omics scientists, bioinformaticians, and biologists, combined with Singapore's multiracial population, the NUS PM TRP is expected to fill a gap in Asia's PM database.

From sample collection, data processing, and results interpretation point of view, this article has also raised some concerns that need to be actively addressed. PM involves a large amount of sample collection. Thus, it is necessary to establish a comprehensive set of rules and laws to protect the rights and interests of the participants. A complete and standardized sample collection, data processing, and results interpretation system also needs to be established to effectively promote the development of PM. Last but not least, a lot more effort needs to go into the mechanistic interpretation of all the data collected, including the meaning of variants and molecules unique to specific populations so that PM outputs such as predictive polygenic risk score (PRS) for disease(s) and targeted clinical interventions can be implemented in time to come.

## Data Availability Statement

The original contributions presented in the study are included in the article. Further inquiries can be directed to the corresponding author.

## Author Contributions

AT: conceptualization and supervision. DK: writing original draft. DK, HY, XS, KW, ET, MW, and AT: review and editing. All authors contributed to the article and approved the submitted version.

## Funding

DK was supported by the NUS Research Scholarship. AT was supported by IMCB, A^*^STAR, Lee Foundation Grant SHTX/LFG/002/2018, FY2019 SingHealth Duke-NUS Surgery Academic Clinical Programme Research Support Programme Grant, Precision Medicine and Personalized Therapeutics Joint Research Grant 2019, Industry Alignment Fund – Industry Collaboration Project (IAF-ICP) I1901E0049, the 2nd A^*^STAR-AMED Joint Grant Call 192B9002, NUHSRO/2021/035/NUSMed/04/NUS-IMCB Joint Lab/LOA, Paris-NUS 2021-06-R/UP-NUS (ANR-18-IDEX-0001), OFIRG21jun-0097, CSASI21jun-0006 and MTCIRG21-0071.

## Conflict of Interest

AT is a co-founder of BetaLife Pte Ltd., but he is not employed by BetaLife Pte Ltd. The remaining authors declare that the research was conducted in the absence of any commercial or financial relationships that could be construed as a potential conflict of interest.

## Publisher's Note

All claims expressed in this article are solely those of the authors and do not necessarily represent those of their affiliated organizations, or those of the publisher, the editors and the reviewers. Any product that may be evaluated in this article, or claim that may be made by its manufacturer, is not guaranteed or endorsed by the publisher.
